# Unfavorable Mortality-To-Incidence Ratio of Lung Cancer Is Associated with Health Care Disparity

**DOI:** 10.3390/ijerph15122889

**Published:** 2018-12-17

**Authors:** Cheng-Yu Huang, Kwong-Kwok Au, Sung-Lang Chen, Shao-Chuan Wang, Chi-Yu Liao, Hui-Hsiang Hsu, Wen-Wei Sung, Yao-Chen Wang

**Affiliations:** 1 Department of Urology, National Taiwan University Hospital, Taipei 10002, Taiwan; bbktony03331@gmail.com; 2 Division of Thoracic Surgery, Department of Surgery, Chung Shan Medical University Hospital, Taichung 40201, Taiwan; Speedmoment@hotmail.com; 3 Department of Urology, Chung Shan Medical University Hospital, Taichung 40201, Taiwan; cshy650@csh.org.tw (S.-L.C.); rosenbeck.wang@gmail.com (S.-C.W.); 4 School of Medicine, Chung Shan Medical University, Taichung 40201, Taiwan; 5 Institute of Medicine, Chung Shan Medical University, Taichung 40201, Taiwan; 6 Department of Surgery, Chung Shan Medical University Hospital, Taichung 40201, Taiwan; Channelb622@gmail.com; 7 Department of Medical Education, Chung Shan Medical University Hospital, Taichung 40201, Taiwan; C91172006@gmail.com; 8 Department of Internal Medicine, Chung Shan Medical University Hospital, Taichung 40201, Taiwan

**Keywords:** lung cancer, mortality, incidence, mortality-to-incidence ratio

## Abstract

The mortality-to-incidence ratio (MIR) is associated with the clinical outcome of cancer treatment. For several cancers, countries with relatively good health care systems have favorable MIRs. However, the association between lung cancer MIR and health care expenditures or rankings has not been evaluated. We used linear regression to analyze the correlation between lung cancer MIRs and the total expenditures on health/gross domestic product (e/GDP) and the World Health Organization (WHO) rankings. We included 57 countries, for which data of adequate quality were available, and we found high rates of incidence and mortality but low MIRs in more developed regions. Among the continents, North America had the highest rates of incidence and mortality, whereas the highest MIRs were in Africa, Asia, Latin America, and the Caribbean. Globally, favorable MIRs correlated with high e/GDP and good WHO ranking (regression coefficient, −0.014 and 0.001; *p* = 0.004, and *p* = 0.014, respectively). In conclusion, the MIR for lung cancer in different countries varies with the expenditure on health care and health system rankings.

## 1. Introduction

Lung cancer is recognized as the leading cause of cancer deaths around the globe [1]. In the USA, it accounts for 27% of cancer-related mortalities, killing more than 85,000 victims in a single year [2]. Part of lung cancer lethality is a result of its late diagnosis, as over half of lung cancer cases are diagnosed with distant metastasis [1]. Therefore, the importance of screening for early stages of lung cancer cannot be overemphasized. Since the 1990s, clinical trials and research on lung cancer screening have flourished [3], and several guidelines have also been established for effective screening [4]. However, screening efficacy in different countries or regions is seldom compared, and screening studies often fail to use credible tools. By contrast, smoking cessation programs unequivocally contribute to reduced cancer risk and improved prognosis in lung cancer patients [5,6,7]. Regardless, the institution of these favorable policies relies on a good health care system.

One innovative parameter for evaluating the prognosis of a specific disease is the mortality-to-incidence ratio (MIR). The value of one-(MIR) also approximates the five-year survival for most cancers [8]. Parkin and Bray showed the significance of the MIR for the completeness of cancer registries in terms of the quality of cancer care [9]. In 2015, Sunkara et al. found a positive correlation between the MIRs for colorectal cancer in different countries and those countries’ health care system rankings, indicating that the MIR could be a potential parameter for evaluating cancer surveillance programs [10]. Overall, the MIR value for a given country depends on that country’s screening policies, which are limited in turn by the state of the country’s health care system.

Screening for colorectal cancer is much simpler and less costly than screening for lung cancer; a much deadlier disease. Therefore, the aim of our study was to determine whether the MIR for lung cancer also shows a relationship with parameters such as total expenditures on health/gross domestic product (e/GDP) and the World Health Organization (WHO) rankings among different countries, regions, and continents.

## 2. Methods

Data acquisition was described previously [11,12,13]. In brief, cancer incidence and mortality data including the numbers, crude rates, and age-standardized rates (ASR) were obtained from the GLOBOCAN 2012 database (http://globocan.iarc.fr/Default.aspx), which is maintained by the International Agency for Research on Cancer, IARC (https://www.iarc.fr/). The definitions are described in the IARC website (http://www-dep.iarc.fr/WHOdb/glossary.htm). The WHO rankings were obtained from the World’s Health Systems of WHO. Health expenditures and life expectancies were obtained from the World Health Statistics of WHO (https://www.who.int/gho/publications/world_health_statistics/en/).

The GLOBOCAN 2012 database contains data from 184 countries. We excluded countries that lacked WHO ranking data (22 countries), or those that had a low availability level of the data (ranking E to G for incidence or ranking 4 to 6 for mortality; 105 countries). Ultimately, 57 countries were included in the analyses. The MIR was defined previously as the ratio of the crude rate of mortality to the incidence [10,11,12,13].

The statistical analysis methods were described previously [11,12,13]. We evaluated the association between the MIRs and variants by linear regression and bivariate correlation using the Spearman correlation test and SPSS statistical software version 15.0 (SPSS, Inc., Chicago, IL, USA). *p* values <0.05 were considered statistically significant. Scatter plots were produced using Microsoft Excel 2010. The data sets analyzed during the current study will be made available upon request.

### Ethics Approval and Consent to Participate

Not applicable. All the data were obtained from the global statistics of GLOBOCAN (http://globocan.iarc.fr/Default.aspx). This is a study of analytic epidemiology, and we did not perform any intervention on human participants. We confirm that this study complied with national guidelines (http://law.moj.gov.tw/LawClass/LawAll.aspx?PCode=L0020162).

## 3. Results

### 3.1. Crude Rates of Incidence/Mortality According to Regions

The crude rates of incidence and mortality for lung cancer in different regions of the world are summarized in Table 1. These regions were grouped by development status, WHO region categories, and continents. When standardized by patient age, the incidence and mortality rates around the world were 23.1 and 19.7, respectively, for a MIR of 0.87. When compared with less developed regions, the more developed regions had much higher crude rates of incidence and mortality (60.9 and 50.3, respectively for more developed vs. 18.4 and 16.6, respectively for less developed). However, the MIR was markedly lower for the more developed regions (0.83 vs. 0.90, respectively). In terms of the WHO region categories, the European region had the highest mortality and incidence crude rates (49.7 and 43.0, respectively), while the African region had the lowest (2.1 and 1.7, respectively). The region with the lowest MIR was the Americas (0.81), and that with the highest was Southeast-Asia (0.91). When categorized by continents, Africa had the lowest incidence and crude mortality rates (2.8 and 2.5, respectively), while North America had the highest (68.4 and 53.5, respectively). Three continents shared the highest MIR of 0.89 (Africa, Latin-America, and the Caribbean), while Oceania had the lowest MIR (0.75).

### 3.2. Crude rates of Incidence/Mortality and Case Numbers of Incidence/Mortality According to Countries

Table 2 summarizes the lung cancer crude rates of incidence and mortality according to countries. The five counties with a crude rate of incidence greater than 70 were Japan (75.0), the Netherlands (71.6), Belgium (72.2), Canada (73.5), and Denmark (81.6). Three of these five countries were also among those with a crude mortality rate over 60, with Japan (59.4) and Canada (58.0) as exceptions. The other country with a mortality rate over 60 was Poland (61.0).

### 3.3. MIRs According to Countries

The calculated MIRs are also presented in Table 2. The three countries with MIRs over 1.00 were Mauritius (1.37), Estonia (1.05), and Ecuador (1.01). Countries with the lowest MIRs, below 0.75, were Switzerland (0.75), Australia (0.73), and Costa Rica (0.70).

### 3.4. The Association between WHO Ranking, e/GDP, and MIR among Countries

**Figure 1:**
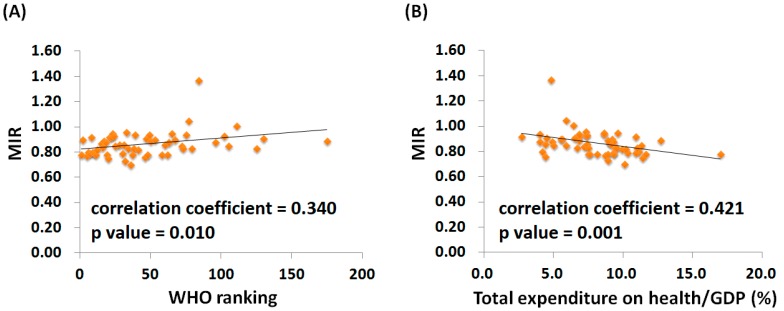
The (**A**) World Health Organization rankings and (**B**) total expenditures on health/GDP were associated with the mortality-to-incidence ratio (MIR) for lung cancer. The *p* value was determined by bivariate correlation using the Spearman correlation test. The country with an extreme value is Mauritius (MIR = 1.38).

Supplementary Figures S1 and S2 show the linear correlation between the WHO ranking, e/GDP, and the crude and age-standardized mortality and incidence rates for different countries. When standardized by age, the crude incidence rates maintained a correlation with WHO ranking, whereas the correlation that was evident between the mortality rate and WHO ranking before standardization disappeared after standardization. Conversely, the positive correlation between e/GDP, crude incidence, and crude mortality rates for different countries remained significant even after standardization. Figure 1 shows the scatter plot for MIR, WHO ranking (Figure 1A), and e/GDP (Figure 1B) among the different countries. We found a positive correlation between the MIRs and WHO rankings (regression coefficient: 0.001; 95% confidence interval: <0.001 to 0.002; R^2^ = 0.14, *p* value = 0.014; for the Spearman correlation test: Correlation coefficient = 0.340, *p* value = 0.010), indicating that a better healthcare ranking led to a lower MIR. Linear regression and bivariate correlation using the Spearman correlation test also indicated a correlation between the MIRs and e/GDP (regression coefficient: −0.014; 95% confidence interval: −0.024 to −0.005; R^2^ = 0.144, *p* value = 0.004; for the Spearman correlation test: Correlation coefficient = 0.421, *p* value = 0.001). The MIRs were better for countries with higher e/GDP.

## 4. Discussion

We found a significant correlation between the WHO rankings and the MIR for lung cancer in different countries. In general, the MIR decreased as the ranking improved. Therefore, although lung cancer and colon cancer differ in their characteristics, the negative correlation found by Sunkara et al. for MIR and colon cancer is still applicable to lung cancer [10]. This consistent correlation could reflect the abundance of tools now available for lung cancer screening in countries with better health care rankings.

Low dose computed tomography (LDCT) has now become a vital component in lung cancer screening, as detection of non-calcified lung nodules is ten times more frequent with LDCT than with chest X-rays [14]. The use of LDCT in a national lung screening trial also resulted in a significant increase in the incidence rate, and a 20% reduction in the mortality rate when compared to chest X-ray screening [15]. Another powerful and novel screening alternative is the use of molecular biomarkers for screening to detect early stage cases for timely treatment [16]. These relatively new and more effective screening methods are more readily available in countries with better healthcare rankings. Early detection, in combination with effective treatment, brings about the association we have demonstrated here. The association of MIR with eGDP is compatible with that of the WHO healthcare ranking. This is understandable, since a fair financial contribution is an important element of the ranking system.

Public health policy also plays an important role in disease prevention and would contribute to the correlation between the WHO rankings and the lung cancer MIR. For example, smoking cessation has substantial general health benefits, including reduction of cancer incidence and mortality. With respect to lung cancer specifically, smoking is the primary causal factor, and cessation clearly and unequivocally reduces the risk of lung cancer [6]. In a historical cohort studied over 33 years, smoking cessation sharply reduced lung cancer mortality in lung cancer patients [7]. Consequently, quitting smoking and offering smoking cessation interventions are recommended [5]. However, financial and health care system support are necessary for smoking cessation programs, so countries with better WHO rankings would again be expected to have favorable MIRs for lung cancer, as demonstrated here.

The association between the crude mortality rate and the WHO ranking is somewhat disturbing. However, this association is expected, since we used the crude mortality rate instead of the disease mortality rate. The higher incidence rates in better-ranked countries result in more lung cancer cases and higher crude mortality rates. Age may have also been a cause of the outcome, since people in countries with better rankings are more likely to have longer life expectancies than those in countries with worse rankings, and this would again contribute to higher cancer mortalities. This life expectancy effect could also explain the non-significant result with an age-standardized crude mortality rate.

To our knowledge, this is the first article to address the association between the MIR of lung cancer, the WHO ranking, and e/GDP. However, our study also has some limitations. We did not include countries with poor data quality or with no available data quality assessment, which might result in misleading MIRs. The result is incompleteness of the data collection and reduction in the generalizability of the results to, for example, under-represented countries from Africa. Furthermore, we did not record the additional risk factors, such as smoking percentages, asbestos exposures, and particulate matter 2.5 pollution among the different countries. These risk factors may play important roles in determining the incidence and mortality rates among countries and regions [17,18]. We also collected the cross-sectional data for only one year, so these data may not accurately depict the actual trend of the disease and may not allow inference of causality. Another limitation is the use of the WHO ranking; this ranking was established in 2000, so it may not precisely represent the current situation for health care systems among different countries.

Despite these limitations, our study shows higher incidence and mortality rates for lung cancer in more developed regions and in countries with better WHO rankings. The MIRs of different countries were also negatively correlated with the WHO rankings. The MIR is therefore a potentially useful parameter for evaluating the screening and healthcare treatment status for lung cancer.

## 5. Conclusions

The MIR for lung cancer varies with the expenditure on health and the ranking of health systems, making it a potentially useful parameter for global evaluation of the clinical outcomes for lung cancer.

## Figures and Tables

**Table 1:** Summary of the case number, rates, and mortality-to-incidence ratio of the incidence and mortality according to regions in lung cancer.

Region	Number		Crude Rate ^1^		Age-Standardized Rate ^1^		Mortality-to-Incidence Ratio ^2^
	Incidence	Mortality	Incidence	Mortality	Incidence	Mortality	
World	1,824,701	1,589,925	25.9	22.5	23.1	19.7	0.87
Development							
More developed regions	758,214	626,570	60.9	50.3	30.8	24.2	0.83
Less developed regions	1,066,487	963,355	18.4	16.6	20.0	18.0	0.90
WHO region categories							
WHO Africa region	18,051	16,108	2.1	1.8	3.9	3.5	0.86
WHO Americas region	324,301	262,314	34.0	27.5	26.0	20.4	0.81
WHO East Mediterranean region	32,542	28,977	5.2	4.7	7.9	7.1	0.90
WHO Europe region	448,618	388,203	49.7	43.0	28.7	24.0	0.87
WHO South-East Asia region	162,003	146,216	8.7	7.9	10.5	9.5	0.91
WHO Western Pacific region	838,978	747,920	45.5	40.6	32.8	28.5	0.89
Continent							
Africa	30,314	27,083	2.8	2.5	5.0	4.5	0.89
Latin America and Caribbean	84,520	74,602	14.0	12.4	13.7	12.0	0.89
Northern America	239,781	187,712	68.4	53.5	38.3	28.6	0.78
Asia	1,045,695	936,051	24.6	22.0	23.4	20.7	0.89
Europe	410,220	353,848	55.3	47.7	29.0	24.0	0.86
Oceania	14,171	10,629	37.6	28.2	25.3	18.3	0.75

^1^ per 100,000 population; ^2^ the percentage in the ratio of the crude rate of mortalities and the crude rate of incidences.

**Table 2:** Summary of World Health Organization rankings, total expenditure on health/GDP, life expectancy, lung cancer incidence, mortality, and mortality-to-incidence ratio of selected countries.

Country	Ranking	Total Expenditure on Health/GDP (%)	Life Expectancy ^1^	Number		Crude Rate ^2^		Age-Standardized Rate ^2^		Mortality-to-Incidence Ratio ^3^
				Incidence	Mortality	Incidence	Mortality	Incidence	Mortality	
France	1	11.6	82	40,043	31,434	63.1	49.5	35.0	25.3	0.78
Italy	2	9.2	83	37,238	33,531	61.1	55.0	24.5	20.7	0.90
Malta	5	8.7	81	181	139	43.2	33.2	20.4	15.6	0.77
Singapore	6	4.2	83	1974	1590	37.6	30.2	24.9	19.8	0.80
Spain	7	9.3	83	26,715	21,118	57.1	45.2	30.3	22.8	0.79
Oman	8	2.7	76	76	70	2.6	2.4	5.1	4.8	0.92
Austria	9	11.1	81	4576	3658	54.3	43.4	27.5	20.7	0.80
Japan	10	10.3	84	94,855	75,119	75.0	59.4	24.6	17.4	0.79
Norway	11	9.3	82	2845	2219	57.4	44.7	30.0	22.2	0.78
Portugal	12	9.9	81	4192	3441	39.2	32.2	20.2	15.7	0.82
Iceland	15	9.0	82	162	141	49.3	42.9	29.8	24.5	0.87
Luxembourg	16	7.2	82	261	218	49.9	41.7	28.4	23.1	0.84
Netherlands	17	12.7	81	11,968	10,609	71.6	63.5	37.2	30.5	0.89
United Kingdom	18	9.3	81	40,382	35,581	64.3	56.7	30.0	25.4	0.88
Ireland	19	8.9	81	2273	1778	49.6	38.8	31.3	23.6	0.78
Switzerland	20	11.4	83	4237	3194	54.8	41.3	27.3	20.0	0.75
Belgium	21	10.9	80	7794	7179	72.2	66.5	36.8	30.5	0.92
Colombia	22	6.8	78	4780	4417	10.1	9.3	11.0	10.1	0.92
Sweden	23	9.6	82	3891	3695	41.0	38.9	19.1	16.4	0.95
Cyprus	24	7.3	82	276	258	24.4	22.8	16.2	14.7	0.93
Germany	25	11.3	81	50,813	43,420	62.0	53.0	27.5	22.2	0.85
Israel	28	7.4	82	2270	1956	29.5	25.4	21.2	17.9	0.86
Canada	30	10.9	82	25,481	20,108	73.5	58.0	37.9	28.4	0.79
Finland	31	9.1	81	2494	2138	46.2	39.6	20.1	16.7	0.86
Australia	32	8.9	83	11,331	8232	49.4	35.9	27.0	18.5	0.73
Chile	33	7.3	80	3127	2980	17.9	17.1	13.3	12.5	0.96
Denmark	34	11.0	80	4566	3806	81.6	68.1	39.2	31.4	0.83
Costa Rica	36	10.1	79	363	256	7.6	5.3	7.3	5.1	0.70
United States of America	37	17.0	79	214,226	167,545	67.8	53.1	38.4	28.6	0.78
Slovenia	38	9.4	80	1360	1131	66.7	55.4	33.9	26.8	0.83
Cuba	39	8.6	78	6143	5763	54.6	51.2	32.9	30.1	0.94
New Zealand	41	10.2	82	2027	1659	45.4	37.2	25.9	20.8	0.82
Bahrain	46	4.4	77	84	64	6.2	4.7	15.5	12.4	0.76
Thailand	47	4.5	75	19,505	17,669	27.9	25.3	20.9	19.1	0.91
Czech Republic	48	7.5	78	6683	5228	63.3	49.5	32.5	24.7	0.78
Malaysia	49	4.0	74	4403	4134	15.0	14.1	17.9	17.0	0.94
Poland	50	6.8	77	26,230	23,371	68.5	61.0	38.0	33.4	0.89
Jamaica	53	5.6	74	512	460	18.5	16.7	18.2	15.8	0.90
Korea, Republic of	58	7.6	82	22,873	17,848	47.1	36.7	28.7	21.3	0.78
Philippines	60	4.4	69	12,074	10,369	12.5	10.7	19.3	17.0	0.86
Slovakia	62	8.1	76	2531	1981	46.2	36.1	28.3	21.6	0.78
Egypt	63	4.9	71	5017	4488	6.0	5.3	7.2	6.5	0.88
Uruguay	65	8.6	77	1411	1336	41.6	39.4	27.4	25.1	0.95
Trinidad and Tobago	67	5.5	71	184	165	13.6	12.2	12.2	10.8	0.90
Belarus	72	5.0	72	4012	3422	42.1	35.9	26.2	22.1	0.85
Lithuania	73	6.7	74	1555	1292	47.2	39.2	26.2	21.2	0.83
Argentina	75	6.8	76	11,244	10,531	27.3	25.6	20.9	19.1	0.94
Estonia	77	5.9	77	632	665	47.2	49.6	24.4	23.6	1.05
Ukraine	79	7.5	71	17,251	14,270	38.4	31.8	22.2	18.5	0.83
Mauritius	84	4.8	74	149	204	11.3	15.5	9.9	13.4	1.37
Fiji	96	4.0	70	45	39	5.1	4.5	6.0	5.1	0.88
Bulgaria	102	7.4	75	3936	3659	53.2	49.5	28.1	25.9	0.93
Latvia	105	5.9	74	1183	1002	52.9	44.8	27.8	22.2	0.85
Ecuador	111	6.4	76	1035	1057	7.0	7.1	7.2	7.2	1.01
Brazil	125	9.5	75	34,280	28,285	17.3	14.3	16.3	13.3	0.83
Russian Federation	130	6.5	69	55,805	50,888	39.1	35.7	24.0	21.5	0.91
South African Republic	175	8.9	60	7242	6465	14.3	12.7	18.5	16.7	0.89

^1^ year; ^2^ per 100,000 population; ^3^ the percentage in the ratio of the crude rate of mortalities and the crude rate of incidences.

## Supplementary Materials

The following are available online at http://www.mdpi.com/1660-4601/15/12/2889/s1, Figure S1: Countries with good World Health Organization rankings have high crude rates of (A) incidence, and (B) mortality, as well as high age-standardized rates (ASR) of (C) incidence. The association between World Health Organization rankings and the ASR of (D) mortality was not statistically significant, Figure S2: Countries with good World Health Organization rankings have high crude rates of (A) incidence, and (B) mortality, as well as high age-standardized rates (ASR) of (C) incidence, and (D) mortality.

## Abbreviations

e/GDP	Total expenditures on health/Gross Domestic Product
LDCT	Low Dose Computed Tomography
MIR	Mortality-to-Incidence Ratio
WHO	World Health Organization
