# Supplementary material for: Unfavorable Mortality-To-Incidence Ratio of Lung Cancer Is Associated with Health Care Disparity
Source: Int J Environ Res Public Health. 2018 Dec 17;15(12):2889. doi: 10.3390/ijerph15122889 (PMC6313465; doi:10.3390/ijerph15122889)
Supplement: Supplementary file 1 [file ijerph-15-02889-s001.pdf]

Int. J. Environ. Res. Public Health **2018**, *15*, 0000; doi:

Supplementary Materials: Unfavorable Mortality-To-Incidence Ratio of Lung Cancer is Associated with Health Care Disparity

Cheng-Yu Hua, Kwong-Kwok Au, Sung-Lang Chen, Shao-Chuan Wang, Chi-Yu Liao, Hui-Hsiang Hsu, Wen-Wei Sung *and Yao-Chen Wang *

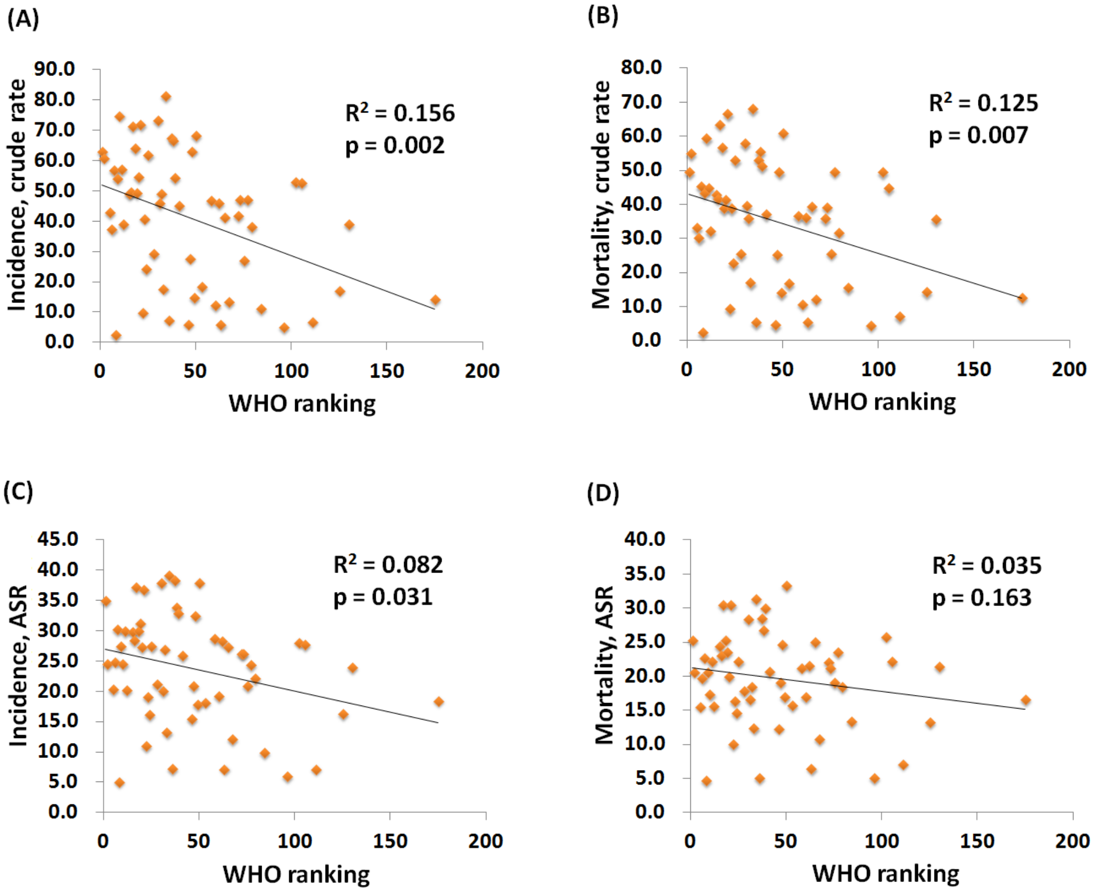

Figure S1. Countries with good World Health Organization rankings have high crude rates of (**A**) incidence, and (**B**) mortality, as well as high age-standardized rates (ASR) of (**C**) incidence. The association between World Health Organization rankings and the ASR of **(D**) mortality was not statistically significant.

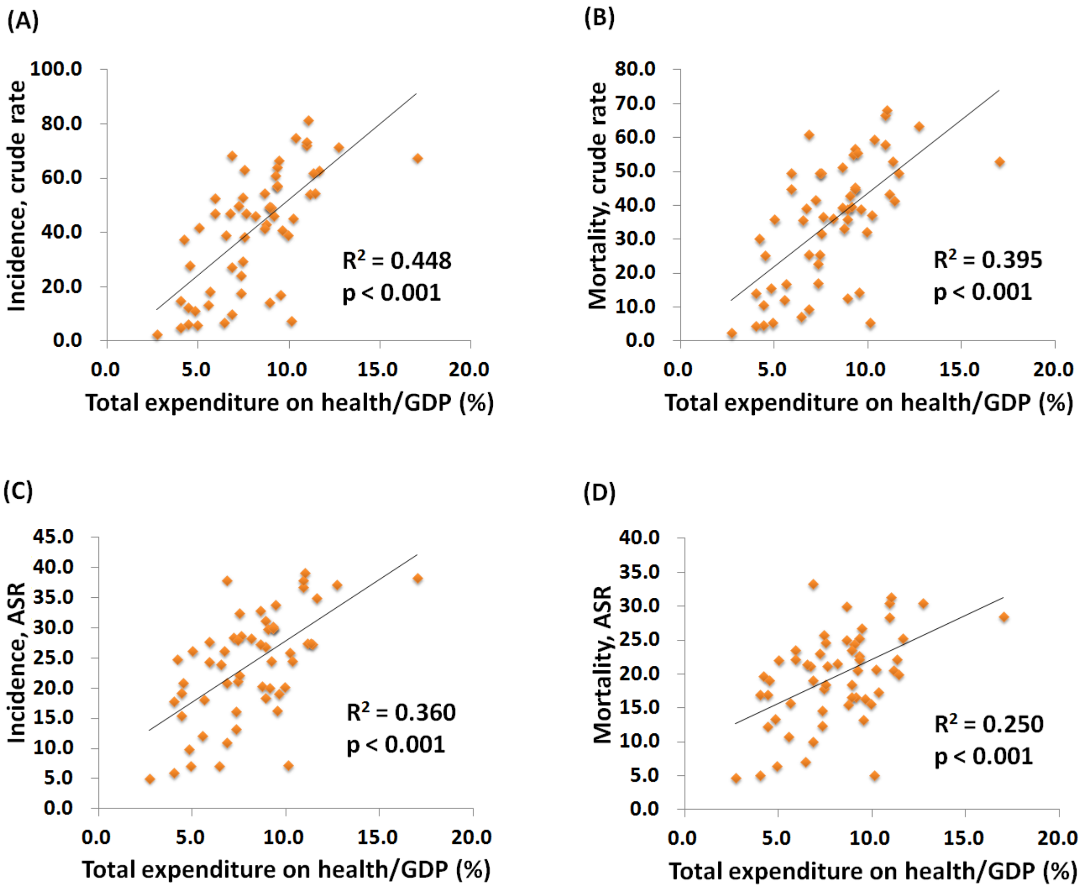

Figure S2. Countries with good World Health Organization rankings have high crude rates of (**A**) incidence, and (**B**) mortality, as well as high age-standardized rates (ASR) of (**C**) incidence, and (**D**) mortality.

© 2018 by the authors. Submitted for possible open access publication under the terms and conditions of the Creative Commons Attribution (CC BY) license (http://creativecommons.org/licenses/by/4.0/).
